# Estimation of the Impact of Meningococcal Serogroup C Universal Vaccination in Italy and Suggestions for the Multicomponent Serogroup B Vaccine Introduction

**DOI:** 10.1155/2015/710656

**Published:** 2015-08-17

**Authors:** Domenico Martinelli, Francesca Fortunato, Maria Giovanna Cappelli, Vanessa Cozza, Maria Chironna, Rosa Prato

**Affiliations:** ^1^Department of Medical and Surgical Sciences, University of Foggia, Via Napoli 20, 71122 Foggia, Italy; ^2^Department of Biomedical Sciences and Human Oncology, University of Bari Aldo Moro, Piazza Giulio Cesare 24, 70124 Bari, Italy

## Abstract

In Italy, the meningococcal C conjugate vaccine (MenC) has been offered in most regions since 2009-2010. The incidence of Invasive Meningococcal Disease (IMD) was 0.25 confirmed cases per 100,000 in 2011, but this may be considerably underestimated due to underdetection and underreporting. This study estimates the impact of the MenC universal vaccination (URV) in the Puglia region by assessing the completeness of three registration sources (notifications, hospitalizations, and laboratory surveillance). Capture-recapture analysis was performed on meningococcal meningitis collected within 2001–2013. The impact of URV among ≤18-year-olds was assessed by attributable benefit, preventable fraction, and prevented fraction. Missed opportunities for vaccination were evaluated from surveillance of IMD. The proportion of detected serogroups was applied to the number of IMD in the postvaccination period to compute the cases still preventable. The sensitivity of the three sources was 36.7% (95% CI: 17.5%–57.9%) and registrations lost nearly 28 cases/year in the period. Attributable benefit of URV was −0.5 cases per 100,000, preventable fraction 19.6%, and prevented fraction 31.3%. Three adolescent cases missed the opportunity to be vaccinated. The multicomponent serogroup B meningococcal vaccine has the potential to further prevent at least three other cases/year. Vaccination strategy against serogroup B together with existing programmes makes IMD a 100% vaccine-preventable disease.

## 1. Background 

*Neisseria meningitidis* is one of the leading causes of bacterial meningitis and sepsis and can also cause pneumonia and other localized infections. Invasive Meningococcal Disease (IMD) is associated with substantial mortality and long-term morbidity worldwide. There are 12 serogroups, but the majority of invasive meningococcal infections are caused by organisms from the A, B, C, X, Y, or W-135 serogroups [1].

Despite significant gaps in data limit description of IMD epidemiology in some parts of the world, it is generally recognized that mass campaigns using conjugated meningococcal vaccines in the last decade have led to the control of serogroup C disease in many developed countries [2].

In Canada, the decline in IMD incidence was at least partly attributable to the universal infant serogroup C conjugate immunization programmes and adolescent catch-up programmes that started as early as 2001-2002 [3, 4]. In the USA, where in 2005 the Advisory Committee on Immunization Practices recommended routine vaccination with the quadrivalent meningococcal conjugate vaccine (MenACWY) for adolescents aged 11–18 years, the incidence of* Neisseria meningitidis* infections mostly decreased from 2006 to 2010 in the targeted population [5, 6].

In Europe, the United Kingdom was the first country to introduce meningococcal serogroup C conjugate vaccine (MenC) in 1999, incorporating it into the routine childhood immunization schedule. In 2000, a catch-up campaign was implemented for adolescents ≤18 years, later extended to young adults up to 24 years of age. As a consequence, in England, hospital admissions decreased from 34.54 per 100,000 children <15 years old in 1999 to 12.40 per 100,000 in 2011 [7].

At a broader level, in 2011, 3,814 confirmed cases of IMD were reported by 29 European Union/European Economic Area (EU/EEA) countries (0.75 per 100,000), mostly in children younger than five years of age (5.73 per 100,000), followed by adolescents and young adults aged 15–24 years (1.29 per 100,000). The majority of cases were due to serogroups B and C, with serogroup B being dominant, mainly attributable to the introduction of the MenC universal routine vaccination (URV) in some EU/EEA countries [8].

In Italy, although only recommended at a national level for at risk groups under the National Immunization Plan 2005–2007, MenC has also been offered to other targets based on individual regional vaccination policies [9]. Since 2009-2010, most regions have established a universal infant free-of-charge programme, most commonly based on active call, reaching an average vaccination coverage of 68% in the 2008 birth cohort [10]. The Puglia region (Southern Italy, with approximately 4,000,000 inhabitants) introduced 1-dose MenC URV for children aged 15 months in 2006 [11], achieving a vaccination coverage higher than 80% (Table 1).

In the same period, 12 regions recommended the immunization of adolescents aged between 11 and 16 years, with one dose [12]. Puglia also introduced the active, free-of-charge offer of 1-dose MenC at 11-12 years of age in 2006 [11] and replaced it with MenACWY in 2012 [13], reaching a vaccination coverage against meningococcus C of nearly 60% (Table 1).

The National Vaccination Plan 2012–2014 included MenC URV in the list of “Essential Health Interventions” for toddlers between 13 and 15 months of age and 11–18-year-old adolescents [14].

IMD is rare in Italy where 0.25 confirmed cases per 100,000 population were observed in 2011, based on surveillance data submitted to The European Surveillance System [8]. Reported incidence, however, may be considerably underestimated due to underdiagnosis (underascertainment) and underreporting affecting IMD surveillance, particularly in some regions [12].

Monitoring the incidence of meningococcal disease is important to evaluate the impact of the implemented vaccination strategies, and to advise on the use of the new multicomponent serogroup B meningococcal (4CMenB) vaccine. This has recently been introduced in Puglia [15] and in other three Italian regions and is under discussion for the introduction on a national scale. This study aims to estimate the impact of MenC URV on the burden of IMD in Puglia by assessing the completeness (sensitivity) of registration systems on meningococcal disease.

## 2. Methods 

### 2.1. Sensitivity Analysis of Data Sources

In Italy, three surveillance sources are available for monitoring meningococcal disease:

- *Mandatory Notification System* (referred to as the Sistema Informativo delle Malattie Infettive (SIMI)), implemented in 1990 by the Ministry of Health, according to which physicians have to report every case of meningococcal meningitis (International Classification of Diseases, Ninth Revision (ICD9) code: 036.0). The notification database contains: a unique patient number, date of birth, gender, first and last name, postal code, municipality, date of notification, date of first symptoms, and date of diagnosis.
- *National Surveillance of Invasive Bacterial Diseases* (referred to as MIB), implemented in 1994 by the Istituto Superiore di Sanità (ISS), with the aim of collecting data on the type of* Neisseria* causing invasive disease from blood and/or cerebrospinal fluid (CSF) isolates. This source contains: a unique patient number, date of birth, gender, first and last name, municipality, laboratory that submitted the strain, date of collection and receipt of the sample, and typing results.
- *Hospital Discharge Registry* (HDR), where IMD is identified by the International Classification of Diseases, Ninth Revision, Clinical Modification (ICD9-CM) codes 036.xx as main or secondary diagnosis. Data in this source are: a personal ID number, first and last name, date of birth, gender, postal code, municipality, date of hospital admission and discharge, ICD9-CM codes, and outcome (deceased or not).

To evaluate the completeness of these three sources in describing IMD incidence, a capture-recapture analysis, similar to that proposed by de Greeff et al. in 2006 [16], was performed on data collected between 2001 and 2013 in Puglia, assuming that the region had a closed population in the considered years (0.6% increase, data from Italy's National Census Bureau (ISTAT) estimate of native- and foreign-born [17]; first assumption). To ensure that each individual had the same chance of being included in all three sources [18], the evaluation was restricted to meningococcal meningitis (ICD9-CM 036.0), as meningococcal sepsis or other clinical pictures of IMD are not notifiable by law in Italy [19] (second assumption). For the capture-recapture analysis involving three or more sources, the independence assumption (third assumption) was not crucial because interaction terms can be incorporated into regression models to adjust for source dependencies [20]. The homogeneity of capture (fourth assumption) was directly fulfilled by the linkage of records between the three sources by three patient variables: first name, last name, and date of birth. The three-source analysis was performed by fitting eight log-linear models using STATA's user-written program “recap” module, providing standard three-source capture-recapture analyses without covariates [21]. The population size (that is the total number of cases, including the number of cases not registered in any of the three sources) and the Confidence Interval estimates were computed according to a goodness-of-fit based method proposed by Regal and Hook [22]. The choice of the final model was based on the likelihood ratio test statistic (*G* ^2^), the Akaike Information Criterion (AIC), and the Bayesian Information Criterion (BIC) [23, 24]. The best-fitting model was the one with the lowest* G* ^2^, AIC, and BIC [25].

Sensitivity of each source was estimated by dividing the observed number of cases in each source by the capture-recapture estimate of the total population [16]; overall sensitivity of the three surveillance registries was computed by dividing the number of cases registered in at least one of the three sources by the capture-recapture estimate of the total population.

### 2.2. Impact of Meningococcal URV on the Burden of IMD

Record linkage in accordance with the above-mentioned design was performed to obtain the number of IMD registered in at least one of the three sources (pool of cases). Crude and age-adjusted annual incidence rates were calculated by dividing the pool of cases by the number of residents in Puglia and applying the region's age-specific rates to the Italian 2001–2013 standard population, respectively (data from Italy's National Census Bureau estimate) [17]. Data were compared to the national hospitalization rates [10]. Crude and age-specific incidence rates before the introduction of URV (calculated as the average annual rates between 2001 and 2005, prevaccination period) were compared to the average annual rates within 2006–2013 (postvaccination period) by calculating the Incidence Rate Ratios (IRRs) with 95% Confidence Interval (95% CI), by using Poisson regression models.

The impact of the vaccination programme was also assessed in the target population, considering subjects ≤18 years of age resident in Puglia, by the following measures:

(i) The* attributable benefit*, that is the reduction in incidence of the disease among vaccinated individuals attributable to the introduction of URV in 2006 [26], calculated as
  (1) $$\begin{array}{c} A_{le}B=I_{v+}-I_{v-}=I_{2006\text{–}2013}-I_{2001\text{–}2005}. \end{array}$$
(ii) The* preventable fraction*, that is the proportion of the disease that would be prevented if the whole population was vaccinated, calculated as
  (2) $$\begin{array}{c} P_{le}F=\frac{I_{p}-I_{v}}{I_{p}}, \end{array}$$
 where *I*_*p*_ was the incidence rate in the population and *I*_*v*_ was the incidence rate in the vaccinated people [26]. Considering the introduction of URV in 2006, the formula was computed as
  (3) $$\begin{array}{c} P_{le}F=\frac{I_{2001\text{–}2013}-I_{2006\text{–}2013}}{I_{2001\text{–}2013}}. \end{array}$$
(iii) The* prevented fraction*, that is the proportion of hypothetical total cases that were prevented by the introduction of URV, calculated as
  (4) $$\begin{array}{c} P_{ed}F=\frac{I_{u}-I_{p}}{I_{u}}, \end{array}$$
 where *I*_*p*_ was the incidence of the disease in the population and *I*_*u*_ was the rate among unvaccinated people [26]. The formula was computed as
  (5) $$\begin{array}{c} P_{ed}F=\frac{I_{2001\text{–}2005}-I_{2001\text{–}2013}}{I_{2001\text{–}2005}}. \end{array}$$
 The prevented fraction could also be calculated as
  (6) $$\begin{array}{c} P_{ed}F=P_{p}\times \left(\;1-RR\;\right), \end{array}$$
 where *P*_*p*_ was the prevalence of subjects protected by the vaccination [26]. In this study, the prevalence of protected subjects was computed as
  (7) $$\begin{array}{c} P_{p}=VC\times VE, \end{array}$$
 where VC was the vaccination coverage reached in the target population and VE was the vaccine efficacy reported for the marketed vaccines [27]. The Relative Risk was
  (8) $$\begin{array}{c} RR=\frac{I_{2006\text{–}2013}}{I_{2001\text{–}2005}}. \end{array}$$

### 2.3. Missed Opportunities in the Meningococcal Vaccination Programme

To review the missed opportunities for vaccination occurring in the meningococcal URV programme, data from an* ad hoc* surveillance system on IMD cases was evaluated.

A prospective population-based, laboratory-confirmed surveillance of possible IMD cases started in Puglia in January 2013 with the aim of describing the epidemiology of IMD in the most affected age groups (0–30 years, residents) over a two-year period. The surveillance network included Infectious Disease and Intensive Care Divisions of all hospitals in the region and the Reference Laboratory for Invasive Bacterial Diseases. Subjects admitted to the participating wards and meeting the clinical case definition for IMD set out by the EU Commission Decision 28/IV/2008 were enrolled as possible cases. This included any person with at least one of the following five clinical signs: fever, meningeal signs, petechial rash, septic shock, and septic arthritis. A confirmed case was any person meeting at least one of the following four laboratory criteria: (i) isolation of* Neisseria meningitidis* from a normally sterile site, including purpuric skin lesions; (ii) detection of* Neisseria meningitidis* nucleic acid from a normally sterile site, including purpuric skin lesions; (iii) detection of* Neisseria meningitidis* antigen in CSF; (iv) detection of gram negative stained diplococcus in CSF [28].

From all specimens taken for routine diagnostic ascertainment within 24 h of enrolment of a possible case, an aliquot was stored at −20°C and sent to the Regional Reference Laboratory for standardized testing of* N. meningitidis* by RT-PCR and multiplex sequential PCR.

For each enrolled subject, physicians involved in the surveillance network collected the following information in an electronic case report form:

- clinical symptoms, date and time of presentation,
- demographics and immunization history for meningococcal vaccines (number of doses, date of vaccination, etc., later validated by the regional immunization registry),
- risk factors and comorbidities,
- data on clinical outcome (recovery, worsening condition, other complications, and death) and hospital resources utilization (length of hospital stay, days in intensive care, daily dosages (DDDs) of antibiotics, and diagnostic tests).

Patients were followed up to 30 days after the beginning of the disease.

The surveillance was conducted in accordance with The Guidelines for Good Clinical Practice and the ethical principles that originate in the Declaration of Helsinki. The protocol was approved by the Institutional Review Board at the Regional Observatory for Epidemiology. For each enrolled subject, written informed consent was obtained from the legal guardians, according to the Italian law. No incentive was provided to encourage study participation.

A missed opportunity was defined as an IMD case occurring in the study period, who was supposed to be vaccinated according to the meningococcal conjugate regional immunization schedule. For each case, the month of disease onset, gender, age, serogroup,* exitus* or sequelae, administered vaccine, and scheduled time of vaccination were reported.

### 2.4. Estimation of Meningococcal URV Further Potential Impact

To estimate the further potential impact of the meningococcal vaccination programme, now including the new 4CMenB vaccine, the distribution of* N. meningitidis* serogroups detected by the MIB surveillance was applied to the total number of cases reported among subjects ≤18 years old in the postvaccination period, in order to compute the annual number of cases that could still be preventable.

For the 4CMenB vaccine, a predicted vaccine strain coverage of 87% was considered [29].

## 3. Results 

### 3.1. Sensitivity Analysis of Data Sources

At a regional level, in the period within 2001–2013,

- 118 cases of meningococcal meningitis were notified to the SIMI,
- 102 cases of meningococcal meningitis were reported to the MIB surveillance,
- 144 hospitalizations for meningococcal meningitis were recorded in the HDR.

Moreover, 873 hospitalizations were coded as meningitis due to unspecified bacterium (ICD9-CM 320.9).

**Figure 1:**
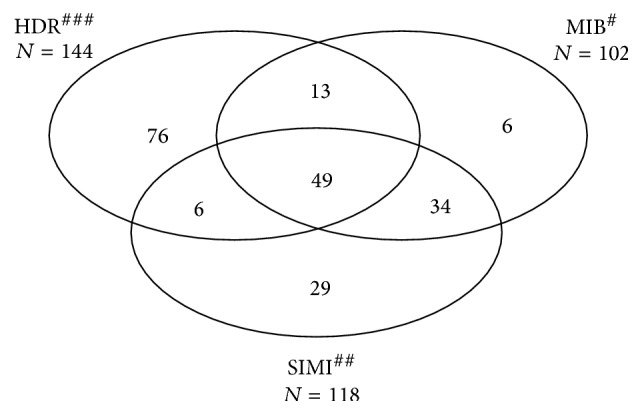
Venn diagram of the number of meningococcal meningitis cases identified by the three sources MIB^#^, SIMI^##^, and HDR^###^ (*N* = 213) in the Puglia region, within 2001–2013. ^#^MIB, Invasive Bacterial Diseases Surveillance; ^##^SIMI, Infectious Diseases Routine Notification System; ^###^HDR, Hospital Discharge Registry.

In the study period, 213 cases of meningococcal meningitis were recorded in at least one of the three sources. Of these, 49 were identified in the three sources, a further 13 were matches between HDR and MIB sources, 34 were matches between MIB and SIMI, and 6 were matches between HDR and SIMI (Figure 1). The log-linear model with the lowest* G* ^2^, AIC, and BIC included two interactions between sources (MIB, SIMI and MIB, HDR) and provided an estimate of 580 (95% CI: 368–1,216) total cases (Table 2).

The overall sensitivity was estimated at 36.7% (95% CI: 17.5%–57.9%). The completeness of HDR was 24.8% (95% CI: 11.8%–39.1%), higher than that of SIMI (20.3%, 95% CI: 9.7%–32.1%) and of MIB (17.6%, 95% CI: 8.4%–27.7%).

### 3.2. Impact of Meningococcal URV on the Burden of IMD

**Figure 2:**
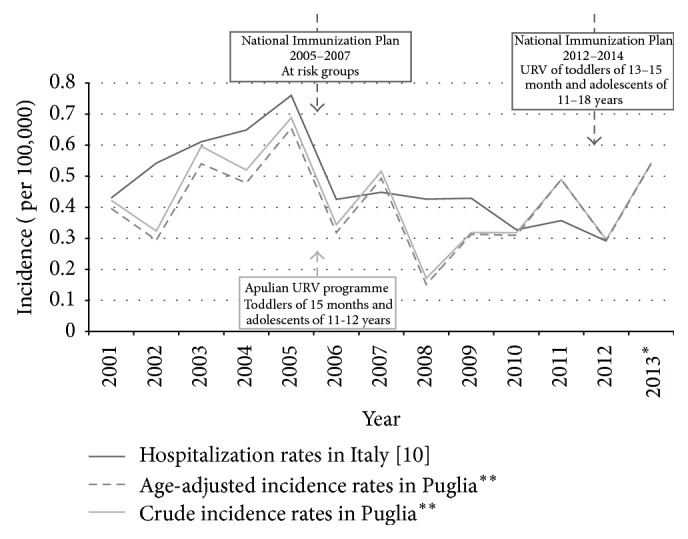
Annual incidence trend of IMD and MenC recommendations in Italy and in the Puglia region, within 2001–2013. ^*∗*^National data not available [10]. ^*∗∗*^Pool of cases from the three sources linkage.

The overall annual incidence trend of IMD showed a sharp reduction immediately after the introduction of the URV in 2006, both in Italy and in the Puglia region (Figure 2).

In the Puglia region, the IRR before and after the introduction of the vaccination programme was 0.7 (95% CI: 0.4–1.4, Table 3). The annual incidence decreased from 1.29 per 100,000 in the prevaccination period to 0.79 per 100,000 in the postvaccination period among subjects ≤18 years of age. The attributable benefit of URV was −0.5 cases per 100,000, while the preventable fraction was 19.6% and the prevented fraction was 31.3%.

**Figure 3:**
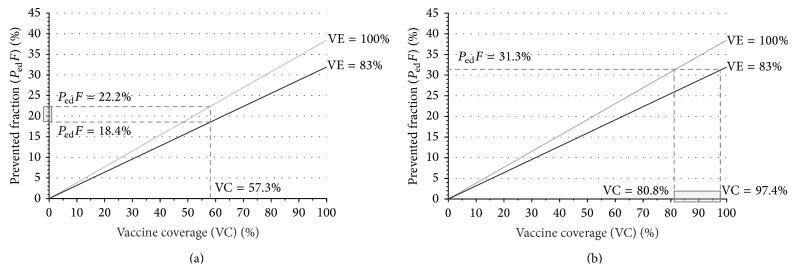
Prevented fraction of IMD cases ≤18 years of age and vaccination coverage against meningococcus C in the Puglia region, within 2001–2013, by vaccine efficacy (VE [27]). (a) Estimated *P*_ed_ *F* for VC of 57.3% among subjects ≤18 years of age, assuming VE of 83–100%. (b) Estimated VC among subjects ≤18 years of age for *P*_ed_ *F* of 31.3%, assuming VE of 83–100%.

Vaccination coverage against meningococcus C among subjects ≤18 years old was 57.3%. Thus, the prevented fraction could be estimated to vary between 18.4% and 22.2% for a VE of 83–100%. (Figure 3(a)). On the other hand, an observed prevented fraction of 31.3% implied that vaccination coverage could range from 80.8% to 97.4% (Figure 3(b)).

### 3.3. Missed Opportunities in the Meningococcal Vaccination Programme

Of 11 confirmed IMD cases among those enrolled ≤30 years of age, three adolescents missed the opportunity to be protected by the vaccination (Table 4).

### 3.4. Estimation of Meningococcal URV Further Potential Impact

Between 2006 and 2013, in the MIB surveillance, serogroup B accounted for 53.8% of isolates, serogroup C for 15.4%, serogroup W for 23.1%, and serogroup Y for 7.7% among subjects ≤18 years of age. Considering an average annual IMD incidence of 0.79 per 100,000 (≈6 cases/year), three cases/year could be attributable to serogroup B, one to group C, and two cases to the other serogroups. Estimating a vaccine strain coverage of 87%, the 4cMenB vaccine has the potential to further prevent at least three other cases/year.

## 4. Discussion

To the best of our knowledge, this is the first study in Italy to provide an assessment of the sensitivity of data sources available for monitoring the incidence of meningococcal meningitis. In some areas, all three registries have the disadvantage of incompleteness due to underdiagnosis (underascertainment), misclassification, and underreporting of IMD [12]. Capture-recapture analysis represents a unique tool to estimate the sensitivity of surveillance registrations and hence the total number of cases [16].

In this study, the completeness of each source alone is no guarantee of an adequate description of disease incidence. Concerning the three linked registries, they are not sufficiently comprehensive in terms of the cases they contain (37%). The number of cases not registered in any of the three sources amounted to 367, meaning that our surveillance systems lost nearly 28 cases/year in the study period. In a similar study by de Greeff et al. in Netherlands, the sensitivity was estimated at 49% for mandatory notifications, 67% for hospital registrations, and 58% for laboratory surveillance [16]. As in our findings, surveillance of meningococcal disease based on hospital admissions seems to capture the most cases, though the data lack serotyping information and are not as timely available as notification data and laboratory surveillance. This seems reasonable as meningococcal disease is so severe that all patients are expected to show up in the hospital [10, 16]. In addition, changes in completeness of registration/reporting by any of the three sources could have affected the results of the capture-recapture analysis, making the interpretation of the chronological trend a challenge.

A significant underreporting affects the Infectious Diseases Routine Notification System in several Italian regions, complicating efforts to understand their occurrence and burden, particularly when the planning and evaluation of vaccination programmes need timely, reliable incidence data. The pattern of this underreporting is a complex mix of factors, including availability and use of appropriate diagnostic services, reporting practices by physicians, and the operation of the surveillance system itself [30]. As regards Invasive Bacterial Diseases, underascertainment remains considerable for the scarce attitude to investigate cases using adequate laboratory tests, as a large number of the discharge records coded as “meningitis due to unspecified bacterium” in this study shows. Real time-PCR is significantly more sensitive than culture, which, so far, has been the most commonly used technique for meningococcal surveillance, leading to an important underestimation of disease burden. Furthermore, it is well known that culture-based methods have an even lower sensitivity compared to molecular methods when the patient has been treated with antibiotics [31]. Real time-PCR also has the advantage of providing diagnosis in the presence of culture-negative samples [32, 33] and timely results.

According to other studies conducted in Italy [10, 34], the incidence rate of IMD decreased after the introduction of the meningococcal URV in 2006, leading to a reduction in the attributable risk among vaccinated individuals. A study by Pascucci et al. in the Emilia Romagna region indicated a 70% decrease in the incidence of meningococcus C-related invasive disease and no cases attributable to serogroup C in children aged 1–4 years after the introduction of the MenC universal vaccination in 2006 [35].

From a public health perspective, it is important to determine the proportion of cases associated with a disease that could be prevented if the target population had received the vaccine for the entire period instead of only a part [36]. In this study, the preventable fraction was 19.6%, meaning that almost one case in five could have theoretically been prevented if URV had been introduced in 2001. The proportion of total cases presumably prevented by the introduction of URV amounted to almost one in three cases (prevented fraction of 31.3%), higher than what could be estimated considering the current coverage against meningococcus C of 57.3% in the target population. Thus, the proportion of subjects protected by the vaccination programme could be higher, up to 80.8–97.4%, due to the indirect herd effect in the unvaccinated population. A nationwide study by Bijlsma et al. in Netherlands has provided further evidence that herd protection, resulting from the reduced carriage of virulent meningococci, was responsible for >36% of MenC vaccine impact [37].

Some missed opportunities occurred in the adolescent meningococcal vaccination programme in Puglia, leading to one death and one case developing long-term sequelae. This highlights the importance of strengthening the ongoing vaccination programme against all preventable serogroups and increasing adequately vaccine timeliness and coverage.

Despite the URV-attributable reduction in the proportion of meningococcal infection due to serogroup C, nearly one case from group C and two cases from groups W and Y could still be preventable every year; cases from serogroup B remain dominant.

Vaccination strategy against serogroup B in infants, now implemented in Puglia, and the existing programmes against serogroups A, C, W, and Y targeting children aged 15 months and 11-12 years old are a full spectrum armoury against all five serogroups, making invasive meningococcal infection a 100% vaccine-preventable disease.

## Figures and Tables

**Table 1:** Vaccination coverage (VC) with meningococcal conjugate vaccines in children ≤24 months and adolescents in Italy and in the Puglia region, within 2006–2013 (postvaccination period), by birth cohort.

Birth cohort	Italy [10]		Puglia region	
	Number of regions providing VC	MenC VC^∗^	MenC VC	MenACWY VC
1995^§^			45.60%	1.80%
1996^§^			51.20%	1.90%
1997^§^			56.20%	2.10%
1998^§^			62.00%	8.50%
1999^§^			46.80%	4.70%
2000^§^			37.30%	21.70%
2001^§^			27.00%	32.30%
2002^§^			24.70%	22.90%
2004^†^			47.00%	
2005^†^	13	48.10%	65.00%	
2006^†^	13	58.50%	74.50%	
2007^†^	16	64.40%	77.90%	
2008^†^	16	68.30%	79.00%	
2009^†^	18	77.80%	81.90%	
2010^†^	18	81.10%	82.40%	
2011^†^			81.10%	

^∗^Average VC in regions which provided data. ^†^One dose of MenC conjugate vaccine at 15 months of age. ^§^One dose of MenC and, since 2012, of MenACWY conjugate vaccine at 11/12 years of age.

**Table 2:** Log-linear models fitted to three sources of data on meningococcal meningitis and the estimated number of cases in the Puglia region, within 2001–2013.

Models	df^∗^	*G* ^2†^	*p* ^‡^	AIC^∗∗^	BIC^∗∗∗^	*x* ^§^	*N* ^¶^	95% CI for *N* ^¶^
Independent (no interaction)	3	114.63	0	108.63	108.79	29	242	229–260
Interaction (MIB^#^, SIMI^##^)	2	21.81	0	17.81	17.92	77	290	260–333
Interaction (MIB, HDR^###^)	2	114.46	0	110.46	110.57	30	243	228–266
Interaction (SIMI, HDR)	2	87.01	0	83.01	83.12	6	219	214–231
**Interaction (MIB, SIMI) and (MIB, HDR)**	**1**	**.58**	**.44**	−**1.42**	−**1.36**	**367**	**580**	**368**–**1,216**
Interaction (MIB, SIMI) and (SIMI, HDR)	1	18.63	0	16.63	16.69	35	248	223–307
Interaction (MIB, HDR) and (SIMI, HDR)	1	84.92	0	82.92	82.97	5	218	213–228
Interaction (MIB, SIMI) and (MIB, HDR) and (SIMI, HDR)	0	0	1	0	0	244	457	271–1,225

^∗^df, degrees of freedom. ^†^ *G* ^2^, likelihood ratio statistic. ^‡^ *p* value for the likelihood ratio statistic. ^∗∗^AIC, Akaike Information Criterion. ^∗∗∗^BIC, Bayesian Information Criterion. ^§^Estimate of the number of cases not reported to any source. ^¶^Estimate of the total number of cases.

^#^MIB, Invasive Bacterial Diseases Surveillance. ^##^SIMI, Infectious Diseases Routine Notification System. ^###^HDR, Hospital Discharge Registry.

**Table 3:** Annual incidence rates per 100,000 Rate Ratios (RRs) and 95% CIs of IMD^∗^ between the prevaccination and the postvaccination period in the Puglia region, within 2001–2013, by class of age.

Class of age	2001–2005		2006–2013		
	*N*	Rate per 100,000	*N*	Rate per 100,000	RR (95% CI)
<1 year	1	2.48	1	2.7	1.1 (0.1–17.1)
1–4 years	3	1.82	1.7	1.1	0.6 (0.1–3.9)
5–9 years	2.2	1.01	1.3	0.6	0.6 (0.1–5.5)
10–14 years	1.8	0.75	1	0.5	0.6 (0.1–7)
15–19 years	2.2	0.86	1.3	0.6	0.6 (0.1–5.5)
20–24 years	2.4	0.84	2.7	1.1	1.2 (0.2–7.2)
25–49 years	3.4	0.23	1.2	0.1	0.3 (0.1–2.8)
≥50 years	4.6	0.34	4.5	0.3	0.9 (0.2–3.3)

Total	**20.6**	**0.51**	**14.7**	**0.4**	**0.7 (0.4–1.4) **

^∗^Pool of cases from the three sources' linkage.

**Table 4:** Missed opportunities in the meningococcal vaccination programme. Active surveillance of IMD cases ≤30-year-olds, in Puglia region, January 2013–September 2014.

Enrolment date	Gender	Age	Serogroup	Sequelae	Exitus	Vaccine to receive	Active call to vaccination
June 2013	M	11	Y	Partial deafness	No	MenACWY	February 2013
October 2013	F	13	C	None	No	MenACWY	January 2011
May 2014	F	18	C	None	Yes	MenC	February 2007
